# AGSE: A Novel Grape Seed Extract Enriched for PP2A Activating Flavonoids That Combats Oxidative Stress and Promotes Skin Health

**DOI:** 10.3390/molecules26216351

**Published:** 2021-10-20

**Authors:** Kristen L. Huber, José R. Fernández, Corey Webb, Karl Rouzard, Jason Healy, Masanori Tamura, Jeffry B. Stock, Maxwell Stock, Eduardo Pérez

**Affiliations:** 1 Research and Development Department, Signum Biosciences, 11 Deer Park Drive Suite 202, Monmouth Junction, NJ 08852, USA; huber.kristen@gmail.com (K.L.H.); jfernandez@signumbio.com (J.R.F.); cwebb@signumbio.com (C.W.); krouzard@signumbio.com (K.R.); jhealy@signumbio.com (J.H.); mtamura@signumbio.com (M.T.); jstock@princeton.edu (J.B.S.); mstock@signumbio.com (M.S.); 2 Department of Molecular Biology, Princeton University, Princeton, NJ 08852, USA

**Keywords:** PP2A, botanical extract, antioxidant, anti-inflammatory, grape seed, cosmetic

## Abstract

Environmental stimuli attack the skin daily resulting in the generation of reactive oxygen species (ROS) and inflammation. One pathway that regulates oxidative stress in skin involves Protein Phosphatase 2A (PP2A), a phosphatase which has been previously linked to Alzheimer’s Disease and aging. Oxidative stress decreases PP2A methylation in normal human dermal fibroblasts (NHDFs). Thus, we hypothesize agents that increase PP2A methylation and activity will promote skin health and combat aging. To discover novel inhibitors of PP2A demethylation activity, we screened a library of 32 natural botanical extracts. We discovered Grape Seed Extract (GSE), which has previously been reported to have several benefits for skin, to be the most potent PP2A demethylating extract. Via several fractionation and extraction steps we developed a novel grape seed extract called Activated Grape Seed Extract (AGSE), which is enriched for PP2A activating flavonoids that increase potency in preventing PP2A demethylation when compared to commercial GSE. We then determined that 1% AGSE and 1% commercial GSE exhibit distinct gene expression profiles when topically applied to a 3D human skin model. To begin to characterize AGSE’s activity, we investigated its antioxidant potential and demonstrate it reduces ROS levels in NHDFs and cell-free assays equal to or better than Vitamin C and E. Moreover, AGSE shows anti-inflammatory properties, dose-dependently inhibiting UVA, UVB and chemical-induced inflammation. These results demonstrate AGSE is a novel, multi-functional extract that modulates methylation levels of PP2A and supports the hypothesis of PP2A as a master regulator for oxidative stress signaling and aging in skin.

## 1. Introduction

Protein phosphatase 2A (PP2A) is a heterotrimeric protein comprised of A (structural), C (catalytic) and B (regulatory) subunits. The C subunit is subject to methylation at its carboxyl terminus, and this modification drives the association of a specific three subunit combination which is necessary for optimal phosphatase activity. PP2A is a master regulator whose critical role and function has been mostly studied in cancer (reviewed in [1]) and tauopathies (reviewed in [2]); however, more recent studies demonstrate this phosphatase also plays a critical role in oxidative stress signaling, inflammation, and barrier function. For example, reactive oxygen species have been shown to inactivate PP2A [3] resulting in activation of NF-κB-mediated pro-inflammatory signaling. In human dermal fibroblasts, oxidative stress has been shown to induce PP2A demethylation, driving the disassociation of the fully active PP2A holoenzyme trimer to the less active dimeric form [4] and PP2A activation has been proposed as a potential therapeutic target for combating oxidative stress [5]. PP2A activation is required for proper epidermal barrier formation during late embryonic development [6]. Conversely, a decrease in PP2A activity has been shown to lead to failure in filaggrin processing, which is essential for epidermal barrier homeostasis [7]. Moreover, ceramides, which play a critical role in skin barrier function have been reported to activate PP2A [8] as has α-tocopherol (Vitamin E), a commonly used topical antioxidant [9]. Altogether, these data suggest that preventing PP2A inactivation by ensuring it remains in a highly methylated, more active state is critical for combating oxidative stress and promoting skin health.

Given the emerging role of PP2A activation in combating aging by promoting both brain and skin health, we wanted to screen a wide range of botanical extracts to determine which ones could regulate PP2A methylation. Once identified, these extracts could then be further fractionated and characterized to uncover the molecules responsible for inhibiting PP2A demethylation. For example, we recently identified chia seeds as a potential natural source for modulating PP2A activity, and subsequently developed a novel chia seed oil extract, enriched for Vitamin F, that improves skin hydration and barrier function when applied topically [10]. By studying and characterizing coffee, we also demonstrated that eicosanoyl-5-hydroxytryptamide (EHT) and coffee extracts enriched for this molecule and other bioactive tryptamides elevate PP2A methylation levels and activity resulting in protection from several diseases of aging such as Alzheimer’s [11,12] and Parkinson’s disease [11,13,14] in in vivo models. Thus, we sought to investigate what other botanical extracts could inhibit PP2A demethylation and promote its activity by screening a variety of different seeds, fruits, roots, leaves and other natural sources.

Here we demonstrate for the first time that several natural extracts regulate PP2A methylation levels, with grape seed extract (GSE) demonstrating the highest potency. We identified several GSE flavonoids as PP2A methylation modulators (~25%), whereas the vast majority (~75%) did not, including resveratrol. We report the identification and characterization of activated grape seed extract (AGSE), a novel grape seed extract enriched for PP2A activating flavonoids that is significantly more potent in inhibiting PP2A demethylation than commercial GSE. Moreover, gene array analysis of AGSE and commercial GSE applied topically to a 3D human skin model demonstrates AGSE outperforms commercial GSE, selectively targeting key extracellular matrix genes, including several collagens and elastin. Lastly, we show AGSE possesses strong antioxidant and anti-inflammatory properties making it an attractive candidate for skin care.

## 2. Results and Discussion

### 2.1. Botanical Extracts as PP2A Demethylation Inhibitors

Levels of phytochemical abundance can vary across different regions of the plant. For this reason, we evaluated ethanolic extracts produced from the seed, fruit, bark, leaves, and root compartments of 32 different botanicals (Table 1). Our results demonstrate that extracts generated from the fruit portion of the botanicals had little to no activity in preventing PP2A demethylation. Conversely, the root, bark, and leaf compartments of the botanicals showed consistent activity modulating PP2A demethylation with potencies ranging from 2–4 µg/mL. However, extracts generated from the seeds of grape, fennel and red raspberry exhibited the highest potencies overall with IC_50_ values of 0.4, 1, and 1 µg/mL, respectively.

To the best of our knowledge, no reports thus far currently link grape, fennel, and/or red raspberry seed extracts to boosting PP2A methylation levels by inhibiting its demethylation. Knowledge to date mainly focuses on the antioxidant and anti-inflammatory properties of these agents. For example, red raspberries affect against UVB is thought to be due to its ROS scavenging properties and ability to modulate a variety of pro-inflammatory modulators [15]. Fennel seeds have also been demonstrated to protect against UVB-induced photoaging of normal human dermal fibroblasts via activation of Nrf2 and inhibition of MAPK pathways [16]. Moreover, extracts produced from grape seeds have been highly explored. In addition to being widely regarded as a strong antioxidant, grape seed extracts have also demonstrated protection from UVA damage [17], wound healing capabilities through triggering a release of vascular endothelial growth factor and increased cell proliferation [18], as well as having chemo-preventive effects [19].

Due to its potency against PP2A demethylation and widely valued research on additional beneficial properties, we sought to evaluate individual components of the grape seed extract which is rich in polyphenols and flavonoids [20,21,22,23]. We purchased and tested 59 components previously found in grape seeds of varying molecular weight and formula to determine if one or more can be responsible for our extract’s effect on PP2A demethylation. Our screening efforts demonstrate that only 14 of the 59 compounds (24%) tested display activity towards preventing PP2A demethylation (Table 2). Quercetagetin, myricetin and sciadopitysin yielded the highest potencies overall with IC_50_ values of 0.5, 0.99 and 1.1 µM respectively. Surprisingly, one of the most well-known grape seed polyphenols, resveratrol, does not function through this PP2A mechanism. Resveratrol’s role in activation of PP2A in Alzheimer’s disease models and cancer cell adhesion studies are thought to be via maintaining protein content through upregulation of protein expression or prevention of degradation [24,25,26] without mention of PP2A’s methylation status. The data shown here supports that resveratrol does not function through protection of PP2A methylation, as is the case for quercetagetin, myricetin and sciadopitysin which have not been previously linked to PP2A’s methylation status.

**Figure 1:**
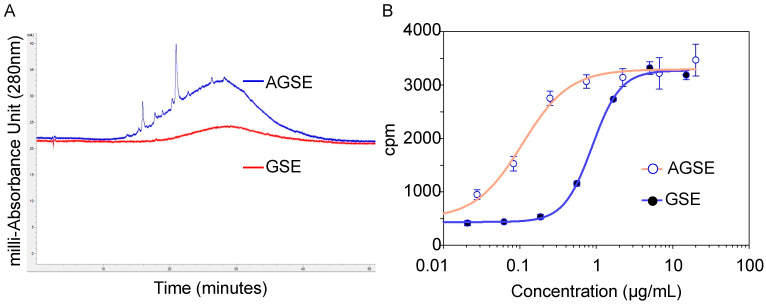
(**A**) HPLC chromatogram of commercial GSE extract yield a PP2A active fraction which was isolated and identified as flavonoid-rich. These fractions yield an “activated” GSE (AGSE) with elevated levels of PP2A-activating flavonoids. (**B**) PP2A demethylation assay demonstrates AGSE is 5-fold more potent than commercial GSE. Data represent the mean ± SD of three independent experiments. The IC_50_ values were determined via the four-parameter logistic curve fit utilizing SigmaPlot Software.

With ~75% of the polyphenols and flavonoids tested having little to no potency towards inhibiting the demethylation of PP2A, we sought to improve the initial grape seed ethanolic extract by developing a process to enrich for these PP2A activating flavonoids to further boost PP2A methylation levels. Through multiple rounds of testing, we identified a novel, activated form of grape seed extract we called AGSE. Chromatographic analysis shows discernable peak enrichment when compared to a commercial grape seed extract (Figure 1A). Furthermore, when tested for its ability to prevent PP2A demethylation, AGSE was found to be ~5-fold more potent than commercial grape seed extract (Figure 1B).

### 2.2. Gene Microarray Analysis of AGSE versus Commercial GSE

**Figure 2:**
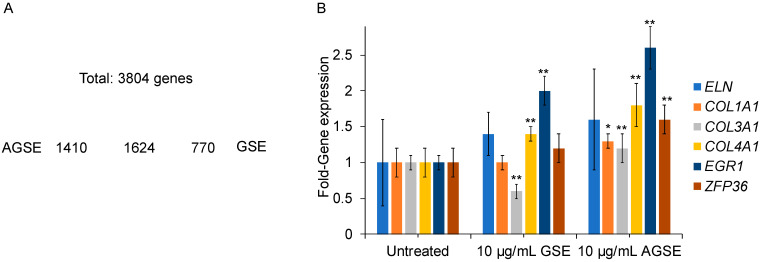
Gene microarray analysis of AGSE versus commercial GSE. (**A**) Gene differential expression was quantified using Clariom™ S assay microarray system method after 24 h incubation in 3D skin model EpiDerm-FT™. AGSE regulated 1410 unique genes compared to commercial GSE. Novel pathways modulated by AGSE were collagen biosynthesis via upregulating collagen types I, III and IV. (**B**) Primary Normal Human Dermal Fibroblasts (NHDFs) were cultured in the presence of each extract for 24 h. Total RNA was collected after 24 h and analyzed by qPCR. GSE and AGSE were tested at 10 μg/mL. Data represents mean ± SE from three independent experiments. * *p* < 0.05; ** *p* ≤ 0.01 relative to vehicle-only treated cells.

Given the different chemical profiles of commercial GSE and AGSE, we sought to characterize the effects of these extracts on skin. Utilizing the human 3D skin tissue model EpiDerm-FT™, commercial GSE and AGSE were applied topically and total RNA was isolated after twenty-four hours to perform gene microarray analysis. Results show that 1% *w*/*v* Commercial GSE regulates 2394 genes (942 upregulated; 1452 down-regulated), while 1% *w*/*v* AGSE modulates over 600 more genes totaling 3034 genes (1122 upregulated; 1912 down-regulated) (Figure 2A). Interestingly, AGSE and Commercial GSE overlap on 1624 regulated genes when compared to untreated skin. Remarkably, our proprietary extract, AGSE was found to regulate 1410 unique genes, almost double the number of unique genes regulated by commercial GSE (770 genes) (Figure 2A).

With the microarray analysis complete, we then investigated specific genes relevant to PP2A activity and skin by utilizing Quantitative PCR (qPCR) and normal human dermal fibroblasts. Effects of natural aging and photoaging on the dermis involves deleterious alterations to the extracellular matrix (ECM) containing collagen [27]. Collagens are essential scaffold proteins that promote skin smoothness and elasticity, but as we age their expression levels (Collagen I and type III) decline [28]. Moreover, Collagen IV is essential for basement membrane stability [29] and is a key ECM protein [30]. Analysis of these key ECM genes suggests that AGSE provides more significant anti-aging benefits to skin than commercial GSE. Results show AGSE boosts Collagen I (*COL1A1*), III (*COL3A1*) and IV (*COL4A1*) by 30%, 20% and 80% respectively. Conversely, commercial GSE has no effect on *COL1A1* (0%), lowers *COL3A1* levels by −40%, and boosts *COL4A1* only by 40%, which is only half that observed for AGSE (Figure 2B). Additionally, while both commercial GSE and AGSE promote elastin (ELN) expression, another ECM protein critical for maintaining skin elasticity and resiliency [31], and Early Growth Response 1 (EGR1), critical for cell growth and wound healing [32], AGSE appears to be more effective. Results demonstrate that AGSE upregulates ELN and EGR1 20% and 60% higher respectively as compared to Commercial GSE (Figure 2B). Moreover, AGSE was also found to upregulate a key PP2A gene ZFP36 ring finger protein (TTP). PP2A modulates phosphorylation of TTP [33] and its activation signals the degradation of pro-inflammatory cytokine mRNAs [34]. Therefore, AGSE increasing TTP expression by 60% (Figure 2B) suggests anti-inflammatory benefits related to PP2A and the TPP mechanism. Altogether, these data demonstrate that AGSE is a novel grape seed extract which exhibits a unique activity profile.

### 2.3. AGSE Possesses Potent Antioxidant Activity

**Figure 3:**
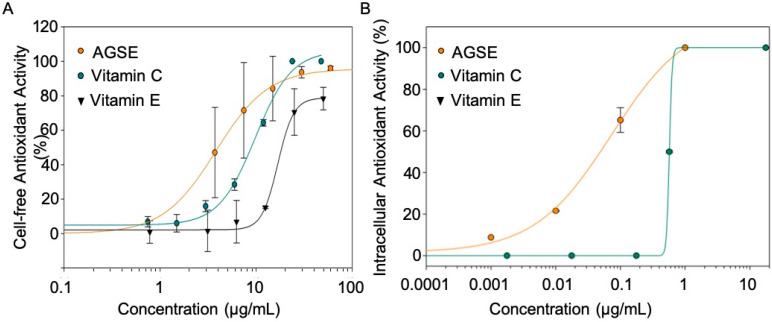
AGSE possesses potent antioxidant activity. (**A**) Cell-free antioxidant capacity of actives were measured via the ABTS assay by metmyoglobin. (**B**) Intracellular antioxidant activity was measured in NHDFs after incubation for 3 h. The activity was determined using DCFH-DA marker of oxidative stress after H_2_O_2_ -induction. The IC_50_ is the concentration of compound producing half maximal inhibition. Data represents mean ± SD from three independent experiments.

The production and release of reactive oxygen species (ROS) is amplified during dermal aging mechanisms and cellular metabolism and thus are a main contributor to the skin aging process [35,36]. Previously, grape seed extracts have demonstrated potent antioxidant activity and have exceled as actives in cosmetic products [37]; therefore, we sought to determine AGSE’s antioxidant properties be testing its activity as a free radical scavenger in both cell-free and cell-based assays. For the cell-free antioxidant test, hydrogen peroxide (H_2_O_2_) was used to induce ROS production. Results show that AGSE inhibits H_2_O_2_-induced free radical ROS production by 100% with an IC_50_ = 3.8 µg/mL, which was ~3 times more potent than Vitamin C (IC_50_ = 10.1 µg/mL) and ~5 times more potent than Vitamin E (IC_50_ = 18.6 µg/mL), both commonly used topical antioxidant actives (Figure 3A).

Several factors including, but not limited to, sun damage, pollution, and chemicals induce intracellular ROS, which overtime can lead to premature cellular aging. If intracellular ROS and other reactive species remain unchecked, they can cause oxidative damage to lipids, nucleic acids and proteins which ultimately accelerates the natural aging process of our skin [38]. However, the onset of oxidative stress can be delayed by functional antioxidant ingredients and extracts that can successfully penetrate dermal cells and scavenge damaging oxidative species. Therefore, we utilized a normal human dermal fibroblast (NHDF) cell-based antioxidant assay for measuring intracellular ROS scavenging capacity. Our results show that AGSE inhibits intracellular oxidative stress by 100% with an IC_50_ = 0.1 µg/mL which once again significantly outperformed Vitamin C (IC_50_ = 0.6 µg/mL) and Vitamin E (IC_50_ = >100 µg/mL), this time 6-fold and at least 1000-fold better respectively (Figure 3B). Altogether, these data suggest that AGSE not only has strong antioxidant properties, but also can successfully penetrate dermal cells and scavenge damaging ROS inside cells.

### 2.4. AGSE Inhibits UV Light and Chemical-Induced Pro-Inflammatory Cytokine Production

**Figure 4:**
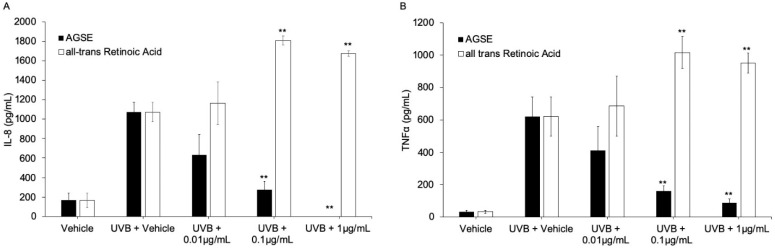
AGSE has anti-inflammatory activity blocking UVB-induced pro-inflammatory cytokine production. Primary Normal Human Epidermal Keratinocytes (NHEKs) were cultured in the presence of AGSE or ATRA (*all-trans* retinoic acid) for 6 h. Later, test compounds were removed and the cells were irradiated with 25 mJ/cm^2^ UVB. Media supernatants were collected after 24 h and analyzed by ELISA for (**A**) Interleukin-6 (IL-8) or (**B**) Tumor necrosis factor alpha (TNFα). Data represents mean ± SE from three independent experiments. ** *p* ≤ 0.01 relative to that in UVB-vehicle irradiated cells.

Ultraviolet light B (UVB) and chemical-induced skin inflammation are mediated through production of inflammatory cytokines via activation of the NFκB pathway [39,40]. We tested AGSE and *all trans* retinoic acid (ATRA), a commonly used anti-aging cosmetic ingredient, to determine anti-cytokine activity by decreasing UVB-induced interleukin-8 (IL-8) and tumor necrosis factor alpha (TNFα) release from cultured primary normal epidermal keratinocytes (NHEKs). Our results show AGSE dose dependently inhibits UVB-induced cytokine release with IC_50_ values = 0.01 and 0.05 µg/mL respectively for pro-inflammatory cytokines IL-8 and TNFα, while ATRA did not have any effect at the highest non-toxic dose tested versus either cytokine (Figure 4; Table 3). A model for chemically induced skin inflammation commonly used to induce cutaneous inflammation is 12-O-tetradecanoyl-phorbol-13-acetate (TPA), as it has been previously shown to increase production of several cytokines in keratinocytes [41,42]. Utilizing this model, our results demonstrate that AGSE effectively blocks TPA-induced release of IL-8 from NHEKs with an IC_50_ = 0.38 µg/mL. Clobetasol, tested as a positive control, expectedly exhibited potent inhibition as well (IC_50_ = 0.50 pg/mL) (Table 3).

UVA-induced photoaging is another means of skin inflammation, mediated through production of inflammatory cytokines, specifically interleukin-6 (IL-6) [43]. We tested AGSE and ATRA to determine anti-photoaging activity by decreasing UVA-induced IL-6 release from cultured primary normal dermal fibroblasts (NHDFs). Our results show AGSE dose dependently inhibits IL-6 release with an IC_50_ = 0.1 µg/mL, while ATRA did not have any effect at the highest non-toxic dose tested (Table 3). These results suggest that while ATRA is an effective anti-aging cosmetic active and has been previously reported to possess anti-inflammatory properties [44], it is not effective at reducing UV-induced inflammation while AGSE is. Given its different antioxidant and anti-inflammatory activity profile, pairing AGSE with ATRA could be an effective combination for promoting healthy, rejuvenated skin.

In summary, we demonstrate here for the first time that PP2A methylation levels are modulated by several different natural extracts, with grape seed extract (GSE) exhibiting the highest potency. With PP2A emerging as an important regulator of oxidative stress, we present here the discovery and development of AGSE, a novel grape seed extract enriched for PP2A-activating flavonoids. Our results show that AGSE not only possesses strong antioxidant and anti-inflammatory properties, but also outperforms commercial GSE in regulating PP2A methylation levels and has a distinct gene microarray profile that selectively targets key extracellular matrix genes including several collagens and elastin. Formulation development of AGSE has been completed and will be tested in the near-term clinically on human subjects to explore and assess its skin benefitting properties when applied topically.

## 3. Materials and Methods

### 3.1. Chemicals and Reagents

Botanical materials were purchased from different sources listed in Table 1. All reagents including compounds in Table 2 were purchased from Sigma-Aldrich Co. (St. Louis, MO, USA). Organic solvents were purchased from Fisher Scientific (Hampton, NH, USA). All chemicals were analyzed by LC/MS (Agilent 1100; Santa Clara, CA, USA), ^1^H and ^13^C NMR (500 MHz and 125 MHz, Bruker, Billerica, MA, USA) for structural identity and confirmed to be >95% pure by analytical HPLC (Agilent 1200; Santa Clara, CA, USA).

### 3.2. Botanical Extraction, Fractionation and AGSE Production

Extracts of seeds, dry fruits, bark, leaves, and root compartments of 32 botanicals were produced by suspending materials in reagent-grade ethanol and heating at 65 °C with stirring for 18 h. The resulting solution was concentrated to dryness on a rotary evaporator in a 30 °C water bath.

Activated grape seed extract (AGSE) was prepared as described in US patent no. 10,912,812 B2. The resulting AGSE material is a glassy, purple/black solid.

### 3.3. Demethylation of PP2A by PME-1 (Protein Phosphatase Methylesterase 1)

[^3^H]-labeled methylated PP2A AC dimer was prepared by incubating PP2A, LCMT1 and [^3^H]-SAM (PerkinElmer; Waltham, MA, USA) in 50 mM MOPS-Na (pH 7.2), 5 mM MgCl_2_, and 1 mM DTT at room temperature for 1 h. Demethylation of PP2A by PME-1 (Protein phosphatase methylesterase 1) was measured using the radioactive filter binding assay format. 20 nM PME-1 was incubated for 15 min with extract or compound, then 20 nM of [^3^H]-labeled methylated PP2A AC dimer was added. Reactions were run at room temperature for 30 min then applied to a 96-well filter plate (MilliporeSigma; Burlington, MA, USA.) containing 30% TCA where proteins were precipitated and separated from the excess of [^3^H]-SAM by washing with 70% ethanol. [^3^H]-incorporation was measured using TopCount NXT scintillation counter (PerkinElmer; Waltham, MA, USA). IC_50_ values were generated from dose–response curves using a four-parameter logistic curve fit in SigmaPlot (Systat Software, Inc., Palo Alto, CA, USA).

### 3.4. Chemical Analysis

The UV absorb fraction of GSE was obtained via preparatory high-performance liquid chromatography using a Reveleris^®^ Prep Purification System (BUCHI Corp.; New Castle, DE, USA). Mobile phases consisted of H_2_O + 0.5% trifluoroacetic acid (TFA) (A) and methanol + 0.5% TFA (B). Separation was performed on a Luna 5 µm C18(2) 100 Å 75 × 30 mm column (Phenomenex; Torrance, CA, USA) and absorbance was monitored at 280 nm. Mass spectrometry (MS) was performed using LTQ Orbitrap MS (ThermoFisher; Waltham, MA, USA). Nuclear Magnetic Resonance (NMR) spectroscopy was recorded on a Bruker 500 MHz spectrometer, dimethyl sulfoxide (DMSO-d6), methanol (CD3OD) or chloroform (CDCl3) was used as NMR solvent.

HPLC was performed using an Agilent 1290 Infinity system. Mobile phases consisted of H_2_O + 2% acetic acid (A) and acetonitrile:methanol (1:1) + 2% acetic acid (B), and the following gradient was used: 5% B for 4 min, 5–80% B over 56 min, 80–95% B over 0.1 min, 95% B for 4.9 min. Separation was performed on a Luna 5 µm C18(2) 100 Å 150 × 4.6 mm column (Phenomenex; Torrance, CA, USA) at a flow rate of 2 mL/min, monitored by absorbance at 510 nm. Samples were prepared as 50 mg/mL solutions in methanol, and 4 uL injections were used for analysis.

### 3.5. Gene Microarray

Differential gene expression analyses were conducted using full-thickness 3D skin equivalents containing epidermal keratinocytes and dermal fibroblasts (MatTek Corp.; Ashland, MA, USA). Treatments (10 mg/mL) were applied to the surface of the skin cultures for 24 h (n = 3). Total RNA was isolated from the skin cultures and global gene expression profiling was analyzed by Affymetrix Human Clariom™ S arrays and data visualized using Transcriptome Analysis Console (TAC) Software (ThermoFisher Scientific, Wilmington, DE, USA). Sample preparation, microarray hybridization, scanning and quality control were carried out at Advanced Biomedical Labs (Cinnaminson, NJ, USA). Significant expression changes between treated and control group (vehicle-only) were filtered using fold change ≥2 (up-regulated) or ≤2 (down-regulated) and *p* Value < 0.05 using empirical Bayes ANOVA. Significant up- and down-regulated genes were analyzed for gene ontology (GO) terms using Metascape [45] website (http://metascape.org/gp/index.html#/main/step1) (accessed on 20 September 2018).

### 3.6. Antioxidant Assays

Radical scavenging antioxidant assay was estimated using the colorimetric antioxidant assay kit obtained from Cayman Chemical Company (Ann Arbor, MI, USA). ABTS (2, 2′-Azino-bis-[3-ehthylbenzthiazoline sulphonate]) was used as the chromogen, which changes into a colored monocation radical form (ABTS^•+^) by metmyoglobin in the presence of hydrogen peroxide, and monitored by measuring absorption at 750 nm using a plate reader (Envision-PerkinElmer; Waltham, MA, USA). Antioxidant inhibition was calculated based on the discoloration of ABTS by serial concentrations of test compounds added simultaneously with myoglobin and hydrogen peroxide. NHDF cells were cultured in DMEM with 10% fetal bovine serum (FBS) and seeded into black wall 96-well plates and incubated for 24 h before treatment. Cells were pre-treated for 3 h with and without test compounds (0.01–3 µM final concentrations) and labeled with 50 µM of dichloro-dihydro-fluorescein diacetate (DCFH-DA). Total fluorescence (Excitation = 485 nm; Emission = 535 nm) was measured after cells were co-incubated for 20 min with 0.1 mM H_2_O_2_ and the test compounds. Cell protection against oxidative stress was calculated using the following formula: Cell protection (%) = [(test compound (%) − hydrogen peroxide-only (%))/(test compound (%) − untreated control (%))] × 100.

### 3.7. Cell Culture

Primary normal human epidermal keratinocytes (NHEKs) and dermal fibroblasts (NHDFs) were purchased from ThermoFisher (Carlsbad, CA, USA) and cultured in EpiLife™ medium (NHEKs) supplemented with Human Keratinocyte Growth Supplement (HKGS) or DMEM (NHDFs) + 10% FBS. All cell types were seeded in plates with growth factor-supplemented medium for 24 h and later media was replaced with growth factor-depleted media for an additional 24 h before treatments and grown at normal conditions (5% CO_2_; 37 °C).

### 3.8. Gene Expression Assays

NHDFs were pre-incubated for 24 h with test materials (1% *v*/*v* ethanol vehicle) in growth factor-depleted fresh media in triplicates. Cells harvested after treatments were homogenized in RNA lysis buffer. Total RNA was extracted using the RNAqueous kit (Thermo Fisher), and cDNA was obtained using the High Capacity RNA-to-cDNA kit (Thermo Fisher). Quantitative PCR (qPCR) was performed using the TaqMan^®^ Fast Advanced Master Mix (Thermo Fisher) and specific TaqMan^®^-probes (*ELN*, Hs00355783_m1; *COL1A1*, Hs00164004_m1; *COL3A1*, Hs00943809_m1; *COL4A1*, Hs00266237_m1; *EGR1*, Hs00152928_m1; *ZFP36*, Hs00185658_m1) for each gene to calculate the relative gene fold expression change per treatment. Gene expression analysis was performed using the comparative Ct method (2^−ΔΔCt^) approach by comparing the Ct values of the treated samples with vehicle-only treated samples and normalized to GAPDH gene expression as endogenous housekeeping gene.

### 3.9. Anti-Inflammatory Assays

Cells were pre-incubated for 2–6 h with test materials (1% *v*/*v* ethanol vehicle) in fresh growth factor-depleted media in triplicates. NHEKs were induced by 5 ng/mL TPA for 24 h or irradiated with 25 mJ/cm^2^ (15 s) broadband 305–12 nm UVB (Daavlin; Bryan, OH, USA) without test materials and incubated for an additional 18 h. NHDFs were irradiated with 12.5 J/cm^2^ UVA (350–12 nm; 40 min) and later incubated without test materials for an additional 18 h. Media supernatants were harvested after induction and used to measure levels of Interleukin-6 (IL-6), Interleukin-8 (IL-8), collagenase (pro-MMP1) or tumor necrosis factor-α (TNF-α) by sandwich ELISA kits (BD Biosciences; San Jose, CA, USA or R&D Systems; Minneapolis, MN, USA). Cells were subjected to viability tests by MTS reduction assay (Promega; Madison, WI, USA).

### 3.10. Statistical Analysis

Statistical significance was determined by ANOVA followed by a Dunnett multiple comparisons test using p-values less than 0.05 as a significant difference. For all biochemical measurements and cytokine levels, samples were assayed in triplicate. Cytokine dose–response curves were generated by fitting data with the Hill, three-parameter equation using the Sigma Plot software, from which the IC_50_ and maximum inhibition were determined.

## Figures and Tables

**Table 1:** Summary results of Botanical extracts in PP2A demethylation activity assay.

Extract Material	Name	Source	IC50 (µg/mL)
Seeds	Almond	Blue Mountain Organics	>5
	Avocado	Farmers Market	6
	Black Raspberry	Fruitsmart/FruitBasics	6
	Blueberry	Fruitsmart/FruitBasics	8
	Celery	Triarco	7
	Coffee	Umalaxmi Organics	6
	Cranberry	Fruitsmart/FruitBasics	10
	Fennel	Triarco	1
	Grape	Fruitsmart	0.4
	Guarana	Pharma Resources International	1.5
	Hazelnut	Bob’s Red Mill	>10
	Red Raspberry	Fruitsmart/FruitBasics	1
Fruits	Avocado	Farmers Market	>25
	Blueberry	Wilderness Family Naturals	>50
	Cranberry	Wilderness Family Naturals	>30
	Grapefruit (Oil)	Organic Herbal Essence	25
	Juniper Berry	Triarco	5
	Maqui Berry	Sunfood Superfoods	>10
	Mulberry	Z Natural Foods	>10
	Promegranate	Navitas	>27
	Schisandra	Triarco	6
	Strawberry	Wilderness Family Naturals	>5
Root, Bark or Leafs	Maca Root	Triarco	3
	Goldenseal Root	Triarco	4
	Magnolia Bark	Triarco	2
	Pygeum Bark	Triarco	3
	Red Raspberry Leaf	Starwest Botanicals	2
Other	Cocoa Butter	Gourmet Imports	12.5
	Cocoa Powder	Gourmet Imports	6.25
	Echinacea Angustifolia	Triarco	4
	Echinacea	Triarco	12

**Table 2:** Summary results of GSE flavonoids in PP2A demethylation activity assay.

Compound	MW (g/mol)	Molecular Formula	IC50 (µM)
2-(acetyloxy)-4-[3,7-bis(acetyloxy)-5-hydroxy-4-oxo-3,4-dihydro-2H-chromen-2-yl]phenyl acetate	472.41	C_23_H_20_O_11_	>15
2′,4-dihydroxy-4′,6′-dimethoxychalcone	300.32	C_14_H_12_O_3_	>25
3,5,7-Trihydroxy-3′,4′,5′-Trimethoxyflavone (Myricetin trimethyl ether)	360.36	C_18_H_16_O_8_	>15
4-methylcatechol	124.14	C_7_H_8_O_2_	>15
5,7-dimethoxy-4′hydroxyflavanone	300.31	C_17_H_16_O_5_	>25
Apigenin	270.25	C_15_H_10_O_5_	>15
Azoxystrobin	403.39	C_22_H_17_N_3_O_5_	>15
Baicalein	270.24	C_15_H_10_O_5_	3.4
Biochanin a	284.27	C_16_H_12_O_5_	~15
Caffeic Acid	180.16	C_9_H_8_O_4_	>15
Catechin	290.28	C_15_H_14_O_6_	>15
Chrysoeriol	300.27	C_16_H_12_O_6_	~25
Cyanidin chloride	322.7	C_15_H_11_O_6_Cl	4.3
Delphinidin	303.246	C_15_H_11_O_7_^+^	5.1
Diadzein	254.24	C_15_H_12_O_4_	>15
Diadzin	416.38	C_21_H_20_O_9_	>15
Diosmin	608.55	C_28_H_32_O_15_	~25
Disometin	300.27	C_16_H_12_O_6_	>25
Epicatechin gallate	442.37	C_22_H_18_O_10_	1.4
Epigallocatechin	306.27	C_15_H_14_O_7_	>15
Epigallocatechin gallate	458.37	C_22_H_18_O_11_	2.6
Eriodictyol	288.26	C_15_H_12_O_6_	>25
Eugenol	164.2	C_10_H_12_O_2_	>15
Ferulic acid	194.19	C_10_H_10_O_4_	>15
Formonoetin	268.26	C_16_H_12_O_4_	>15
Forskolin	410.51	C_22_H_34_O_7_	>25
Gallic acid	170.13	C7H6O5	>15
Gossypetin	318.24	C_15_H_10_O_8_	4.9
Iprodione	330.17	C_13_H_13_Cl_2_N_3_O_3_	>15
Isoorientin	448.38	C_21_H_20_O_11_	>25
Isoquercetin	464.379	C_21_H_20_O_12_	>15
Isorhamnetin (3,5,7,4′-Tetrahydroxy-3′-methoxyflavone)	316.27	C_16_H_12_O_7_	>15
Kaempferide	300.27	C16H12O6	2
Luteolin	286.25	C_15_H_10_O_6_	1.7
Luteolin 7-*O*-B-glucoside	448.38	C_21_H_20_O_11_	>25
Mangostine	410.47	C_24_H_26_O_6_	1.7
Methyl 6,7-dimethoxycoumarin-4-acetate	278.26	C_14_H_14_O_6_	>25
Morin	302.24	C_15_H_10_O_7_	>15
Myclobutanil	288.78	C_15_H_17_ClN_4_	>15
Myricetin	318.25	C_15_H_10_O_8_	0.99
Naringin	580.54	C_27_H_32_O_14_	>25
Narirutin	580.54	C_27_H_32_O_14_	>25
Orientin	448.38	C_21_H_20_O_11_	>25
Phloroglucinol	126.111	C_6_H_6_O_3_	>15
Phosmet	317.32	C_11_H_12_NO_4_PS_2_	>15
Picrotin	310.29	C_15_H_18_O_7_	>25
Procyanidin B1	578.52	C_30_H_26_O_12_	>35
Procyanidin B2	578.52	C_30_H_26_O_12_	>35
Procyanidin B3	578.52	C_30_H_26_O_12_	>35
Pyrogallol	126.11	C_6_H_3_(OH)_3_	~15
Quercetagetin	318.25	C_15_H_10_O_8_	~0.5
Quercetin	302.24	C_15_H_10_O_7_	2.5
Resveratrol	228.25	C_14_H_12_O_3_	>100
Rutin	610.52	C_27_H_30_O_16_	>15
Sciadopitysin	580.547	C_33_H_24_O_10_	1.1
Scutellarin	462.36	C_21_H_18_O_12_	4.9
Syringic acid	198.18	C_9_H_10_O_5_	>15
Tebuconazole	307.82	C_16_H_22_ClN_3_O	>15
Vanillic acid	168.15	C_8_H_8_O_4_	>15

**Table 3:** Summary of inflammatory assay IC_50_ values.

Material	IC_50_ (µg/mL) *			
	NHDFs-UVA-IL-6	NHEK-UVB-IL-8	NHEK-UVB-TNFα	NHEK-TPA-IL-8
Vitamin C	>10	>10	>10	-
Ferulic Acid	0.01	<0.01	<0.01	-
AGSE	0.1	0.01	0.05	0.387
ATRA	>1	>1	>1	-
Clobetasol	-	-	-	5 × 10^−7^

NHDFs, Primary normal human dermal fibroblasts; NHEK, Primary normal human epidermal keratinocytes; UVA, Ultraviolet A rays; UVB, Ultraviolet B rays; IL-6, Interleukin-6; IL-8, Interleukin-8; TNFα, Tumor necrosis factor alpha; AGSE, Activated grape seed extract; ATRA, *all-trans* retinoic acid. * IC_50_ = Inhibitory concentration at 50%. Results represent average cumulative data from 3 independent experiments. IC_50_ values were determined from dose-response curves using a four-parameter logistic curve fit.

## Data Availability

Data available upon request to corresponding author: eperez@signumbio.com.

## Sample Availability

Samples of the extracts or compounds are available from the corresponding author: eperez@signumbio.com.
